# Recent Developments in Biomimetic Antifouling Materials: A Review

**DOI:** 10.3390/biomimetics5040058

**Published:** 2020-10-30

**Authors:** Timothy Sullivan, Irene O’Callaghan

**Affiliations:** 1 School of Biological, Earth & Environmental Sciences, University College Cork, T23 TK30 Cork, Ireland; irene.ocallaghan@ucc.ie; 2 Environmental Research Institute, University College Cork, T23 XE10 Cork, Ireland; 3 School of Chemistry, University College Cork, T12 K8AF Cork, Ireland

**Keywords:** biomimetic, antifouling, bioinspired, biofouling, biofilm, self-cleaning

## Abstract

The term ‘biomimetic’ might be applied to any material or process that in some way reproduces, mimics, or is otherwise inspired by nature. Also variously termed bionic, bioinspired, biological design, or even green design, the idea of adapting or taking inspiration from a natural solution to solve a modern engineering problem has been of scientific interest since it was first proposed in the 1960s. Since then, the concept that natural materials and nature can provide inspiration for incredible breakthroughs and developments in terms of new technologies and entirely new approaches to solving technological problems has become widely accepted. This is very much evident in the fields of materials science, surface science, and coatings. In this review, we survey recent developments (primarily those within the last decade) in biomimetic approaches to antifouling, self-cleaning, or anti-biofilm technologies. We find that this field continues to mature, and emerging novel, biomimetic technologies are present at multiple stages in the development pipeline, with some becoming commercially available. However, we also note that the rate of commercialization of these technologies appears slow compared to the significant research output within the field.

## 1. Introduction

Biomimetics, a term attributed to Otto Schmidt, can be thought of as the study of structure and function in natural systems as inspiration for (sustainable) technological design and engineering [1]. Vincent provides an excellent overview of the development of the field, including the semantics and history of the term [2]. Research into how nature produces materials has seen rapid growth, with the realisation that natural processes can be very efficient, for example biomineralisation [3]. Natural self-assembly processes can also show precise control of surface structure or processes (from the macroscale to the nanoscale) and may also be accompanied by a high fault tolerance. However, as pointed out succinctly by Vincent, it is not sufficient to try and transfer lessons from nature into existing technology, but rather biomimetics hold the promise of more—a new way of looking at the development of technologies, where challenges are approached with an understanding taken from the natural world [2].

The natural world has had, by most accounts, some 3.8 Gyr of diverse development and evolution in order to refine such processes, and the materials subsequently produced [4,5]. It would seem natural to take inspiration from these materials and processes when creating nanomaterials or devices in the lab, perhaps efficiently resulting in novel technologies and approaches; however, it is clear that this path is often not quite as straightforward [2]. Natural processes result in sophisticated structures (often from very simple starting conditions) that utilise complex interplay between surface topography and chemical properties, for example, in order to create multi-functionality. Nano- or micro-scale structures or surfaces that have different length scales superimposed on one another (hierarchical), for example, are common in nature (discussed later) and provide many interesting possibilities when developing novel synthetic materials. As pointed out by Bushan, multifunctionality in natural materials is quite common, and properties such as superhydrophobicity, self-cleaning, drag reduction, thermal insulation, high adhesion strengths or even reversible adhesion, aerodynamic or hydrodynamic lift, incredible mechanical or structural strength (and strength to weight ratios), self-assembly, anti-reflection, structural colour, or self-healing are only some examples of the properties of these materials that are of possible commercial interest [6].

**Figure 1:**
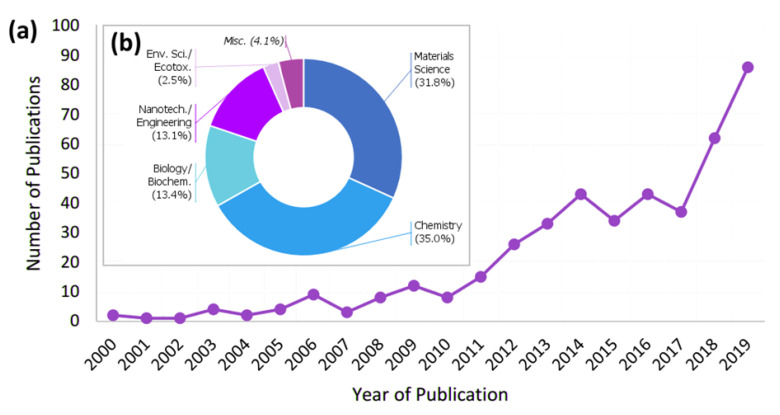
(**a**) An analysis of articles that feature biomimetic or bioinspired antifouling research published per year for the years 2000 until end of 2019 and (**b**) breakdown of source journals by primary field. The search was conducted in April 2020 using both SCOPUS and Web of Science, with the criteria “(biomim * OR bioinsp *) AND (antifoul * OR biofoul *)”. This search shows the increase in the number of publications on biomimetics and antifouling research from a low baseline in 2000 to a greater publication rate in recent years, with almost half of all results returned having been published in the past four years (2016–2019).

There are many excellent published studies and reviews that address the adoption of bioinspired or biomimetic design for these purposes, for example in the field of adhesion studies [1,6,7]. However, existing reviews precede a substantive rise in the number of studies describing biomimetic antifouling strategies. In this review, we emphasise important recent developments in the area of biofouling control and antifouling approaches and focus primarily those that have arisen within the past 10 years. Antifouling or self-cleaning surfaces are also a sub-set of these studies (Figure 1), and the relationship between searching for inspiration from natural adhesives on one hand, and naturally anti-adhesive surfaces or materials on the other hand, is of particular interest. Inspiration may be drawn from organisms, primarily aquatic, that colonise or are subjected to colonisation; the former will develop processes of adhesion, while the latter will develop processes to combat adhesion.

### 1.1. What is Biofouling and Why Control it?

Biofouling can be defined as the *undesired* attachment and growth of life on artificial surfaces [8]. However, there can be great differences in what might be considered as a significant level of biofouling between industries, e.g., in the marine environment, biofouling is often visible to the human eye as mussels, barnacles, or seaweed attached to surfaces such as the hull of a ship or the piling of a pier [9]. Additionally, different applications will impose differing constraints on other physical parameters of the antifouling material. Biofouling and biofilms are present and just as problematic in other industries—for example, in water purification systems, space flight [10], and medicine [11]—but are perhaps less obviously visible and arguably more difficult to control. Flemming provides a sample list of industries known to be affected by biofouling [8].

The common overarching incentives to preventing biofouling in many industries generally relate to either economic or human health impacts, or both—such as cleaning and the associated downtime (e.g., ship fouling and dry docking times [12]), contaminated raw materials, poor performance of critical technologies or components (e.g., heat exchangers or membranes), or shortened component lifetime (of membranes, etc.). Biofouling negatively impacts commercial marine industries and activities, slowing ship speed and increasing the annual fuel consumption of the global shipping fleet [13], and influencing the rate and extent of corrosion on offshore platforms or commercial pipelines for example. Any particular technologies that reduce drag on the hull of a ship in service would have many benefits, increasing fuel efficiency (and aiding in decarbonising global transport) and vessel availability, for example, and reducing invasive species translocation and life-cycle costs [1]. In a medical setting, bacterial biofilms and microbial fouling can pose a serious risk, on medical implants for example, and biofilm development on surfaces can be associated with nosocomial (hospital-acquired) infections [14] and development of antibiotic resistance in clinical or medical settings [15].

The most effective general commercial approaches to preventing biofouling in many settings could have, until recently, been categorised as strategies to attempt to kill and/or clean biofilms. Antifouling or anti-biofilm approaches in many affected industries have largely involved coatings containing biocides as active ingredients to kill or somehow deter fouling [16]. Unfortunately, it is well recognised that these biocidal formulations can have negative effects on the environment [17], and widespread introduction of new biocides has been legislatively regulated in Europe and many areas worldwide as a result of these impacts [18]. This has prompted academic and commercial interest in seeking out new approaches to preventing, reducing, or mitigating the effects of biofouling or biofilm development, particularly in the marine environment, or in membrane technology for water separation, for example [19]. Kyei et al. have recently reviewed the currently available methods for preventing and mitigating growth of algal and other organisms on marine structures in an environmentally friendly and cost-effective (and legal) manner [20].

### 1.2. Biomimetics and Biofouling Control

Biomimetic research and biofouling/antifouling research interests intersect at this point (Figure 1), as some natural biological surfaces are self-cleaning or naturally possess some antifouling capability (or, in the case of living surfaces that prevent colonisation, a capacity to prevent epibiosis). Particularly interesting examples are those chemical defences of marine organisms—many of which are well-known, such as the secondary metabolites of the red seaweed *Delisea pulchra* [21]—that control unwanted epibionts (colonising organisms, or an organism living on another organism). Many of these are still under study [22]. As a recent example, Karnjana et al. have reported that extracts from a red seaweed (*Gracilaria fisheri)* decreased *Vibrio harvey* biofilm formation [23]. The group have subsequently isolated and identified the active compounds [24].

**Figure 2:**
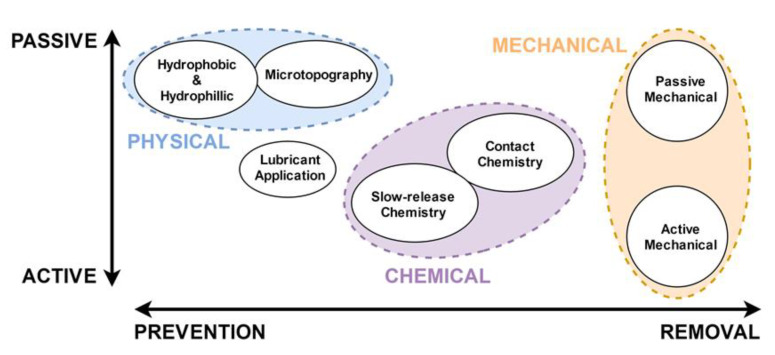
A generalised overview of some of the antifouling strategies that can involve biomimetic aspects or can be inspired by analogous strategies in the natural world. Different strategies can of course be combined, and indeed in many cases can perhaps lead to more efficient or effective fouling control.

Other possible approaches are categorised in general terms in Figure 2 below. These include the well-studied hydrophobic self-cleaning properties of certain plant species, such as the “lotus effect” as observed in *Nelumbo nucifera* (whose mechanisms are elucidated and effects artificially reproduced in [25,26,27]). Some of these approaches have made it out of the laboratory and into broader technical applications, including some interesting recent patents [28] and applications [29]. Of course, these strategies have drawbacks, and there are undoubtedly technical challenges to be overcome in many applications—for example, Flemming points out that the lotus effect, despite its aquatic origins, has not found widespread commercial application on surfaces submerged in water, as the methods to date are based on hydrophobicity requiring the presence of both water and gas phases (a liquid–gas interface), which is difficult to maintain [8]. However, other biomimetic air-retaining strategies such as the “*Salvinia* effect” hold further potential in creating persistent air layers on surfaces such as a ship’s hull [30]. The four criteria that are considered important here for long-term air retention under water are hair-like structures, a hydrophobic chemistry, inclusion of topographic features with undercuts, and the elastic nature of the structures [31]. Novel methods of producing such surfaces that create a “*Salvinia* effect” are currently being examined, including the use of vertically aligned carbon nanotubes [32], and Zhou et al. have recently reported a facile, repeatable method of fabricating *Salvinia*-like surfaces [33].

The potential applications of such materials appear widespread, and Busch et al. recently calculated that surrounding a hull with an air layer could lead to estimated savings of 32.5 million tons of fuel (some 13% of fuel consumption of the worldwide shipping fleet), or USD 18.5 billion and 130 million tons of CO_2e_ per year [34]. Busch et al. also point out that successful application of such air-retaining surfaces could have a combined drag reduction and antifouling effect on a ship’s hull. The authors then provided updated figures incorporating antifouling and drag reduction of 25%, resulting in saving some 40.6 million tons of fuel, or some USD 23.2 billion in cost reduction, or approximately 162.5 million tons of CO_2e_. These figures starkly demonstrate that the potential of such technologies, even if not completely realised in practise, surely justify further investment and exploration of any natural surfaces that achieve such effects.

## 2. Biomimetic Antifouling Strategies

**Figure 3:**
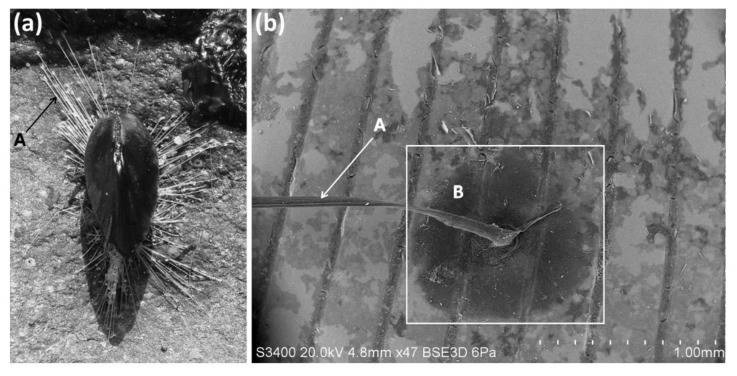
(**a**) An example of an individual marine mussel (centre, *Mytilus* sp.) attached and anchored to a substrate with surrounding (byssus) threads (arrowed, A, top left), and (**b**) a backscattered electron micrograph showing an individual byssus thread in detail (arrowed, A: scale bar = 1 mm) and the accompanying adhesive attachment pad (dark approximately circular region within rectangle, B). In this case, the byssus thread is attached to another bivalve species with a surface texture (the vertical lines within image (**b**)). The extent of the adhesive spreading of the attachment pad is of interest, and the strength of attachment of mussel species makes them particularly prolific biofouling organisms in aquatic environments worldwide. Image (**a**) by Brocken Inaglory from Wikimedia Commons, licensed under CC BY-SA 3.0.

The most pernicious biofouling relies upon the active adhesion of organisms to surfaces at some stage in their life cycle, and, therefore, how (reversible) bonding to a substrate is achieved in an aqueous environment has been of key interest. Perhaps the most notable model systems currently under study are those from the marine environment such as mussel adhesives (Figure 3) or barnacle cements [35,36,37]. Interestingly, this research works in both directions from an applications perspective—research into understanding the biochemistry of adhesion with the aim of developing more effective adhesives in wet environments, and research into anti-adhesive surfaces that can prevent such processes, thus reducing biofouling [38]. Intertidal marine organisms are providing inspiration for the assembly of synthetic molecules into polymeric adhesives [39], but also, as recently pointed out by Almeida et al., the high diversity of invertebrates that inhabit the marine environment has meant that an equally diverse array of structures and principles used in biological adhesives are unexplored [37]. This is perhaps unsurprising, given that nature has provided some fascinating diversity and inspiration in these areas—the enlarged adhesive toe pads of tree frogs, for example, which enable them to climb vertical and overhanging surfaces without appreciable texture or roughness and effectively generate reversible adhesion under many conditions, are of keen interest [40]. These surfaces, which contain nano-topographic structures, can be replicated and are even self-cleaning.

A key objective of antifouling materials or surfaces—particularly those widely associated with mobile platforms and ships in the marine environment—is to create robust coatings that are non-toxic to all (or at least non-target) organisms in use [41]. Examples of such coatings are in development for marine applications and operate on the principle that any shear forces associated with water movement break the adhesive bond between any attached organism and the coating. The coating surface chemistry is engineered so that the adhesive bond between most fouling organisms (ideally all) is weak, and therefore “sloughing off” of fouling happens readily and at low hydrodynamic shear forces (relatively low ship speeds while underway, for example). This is the principle objective of “foul-release” coatings (most of which now contain “lubricant” oils) that are still undergoing development and optimisation for different applications [42,43]. The potential influences of many different aspects of these coatings on biofouling (and foul release) have been examined, including the effects of surface roughness and added texture (topography) [44], the effects of different mechanical properties of coatings including elastic modulus [45], the effects of different chemical additives (especially chemically compatible, but largely biological inert lubricants), and the combined effects of surface energy [46]. There are still some technical challenges to be overcome for these coatings in marine applications, for example, in coating robustness and in development of biofouling while ships are laid up at anchor for extended periods in port.

While optimisation of these coatings continues, the search goes on for inspiration for new technologies, and the surfaces of marine organisms that appear to self-clean are of continued interest here from a biomimetic perspective. If the surface of a species of cetacean, for example, is able to reduce or control fouling (even temporarily), then are there any lessons to be learned that can be used in the design of synthetic materials, and what role do skin or shell surface properties play in this? These questions have led a number of research groups to examine underlying self-cleaning mechanisms in nature and to the natural classification of methods into two categories: self-cleaning with water and self-cleaning without water [47]. Inspiration can also come from the terrestrial sphere and, with a slightly different perspective on anti-adhesive surfaces and the potential for biomimetics in the insect world, Gorb and Gorb have recently provided a fascinating account of wax in plants and the anti-adhesive effects this on insect attachment [48]. How many of the mechanisms presented have potential as possible anti-adhesive technologies or potential antifouling surfaces remains unknown or unexplored—particularly among aquatic insects, for example—and there appears to be a rich vein of inspiration here for potential anti-adhesive formulations and strategies.

### 2.1. Natural Products and Biomimetic Chemistries

Natural surfaces exist that are self-cleaning and/or antifouling—some have the ability to chemically inhibit settlement and growth of colonising organisms on specific surfaces and to self-clean. A common example of these surfaces is the fronds of marine macro-algae (seaweed). These have been carefully examined in the quest to understand natural antifouling solutions [49], perhaps with the ultimate goal of discovering and replicating or synthesising the mechanism(s) responsible. For example, Sánchez-Lozano et al. recently identified five species of macro-algae and two sponge species with a low level of colonizers [50].

Engineered solutions have likewise been presented in the literature. Pan et al. recently tested poly(lactic acid)-based polyurethane with hydrolyzable triisopropylsilyl acrylate side groups, finding that these coatings effectively inhibited the marine bacteria *Pseudomonas* sp. and that marine field tests demonstrated antifouling ability for more than three months [51]. Similarly, Myles et al. tested bioinspired saccharide coatings on 316L grade stainless steel, nylon 6, and poly(ether sulfone), finding that retained biomass was significantly lower on carbohydrate modified samples, suggesting that these types of coatings may have potential applications in marine environments [52].

Multi-faceted approaches that combine chemical and physical methods might be particularly important for marine macro-algae or sponges, where it appears that they must reduce epibiosis in order to remain healthy. Surface sloughing appears to be a relatively common approach in marine macro-algae (in combination with their chemical defences discussed earlier), with recent electron microscopy observations of *Ecklonia maxima* and *Laminaria pallida* by Mayombo et al., for example, confirming that sloughing occurs in both of these kelp species [53]. The authors note here that surface sloughing appears to be an efficient antifouling mechanism for these species, being one of the major factors affecting epiphytic diatom communities, preventing development of the climax stage of community development. Diatoms are a fouling group of interest from a biomimetic perspective in their own right [54], even if they are a particularly difficult biofouling group to control [55], and much work continues on understanding the mechanisms of diatom adhesion [56].

Amongst the sponges, as reported by Barthel and Wolfrath, for example, tissue sloughing can aid in shedding sediment and reducing the settlement of small organisms on sponges [57]. They reported over three decades ago that the sponge *Halichondria panicea* counteracts ostia clogging and the establishment of a surface microfouling community by such sloughing, and also inhibits further fouling development, although less research appears to have been conducted or reported on sloughing in marine sponges in more recent times.

Such natural strategies have, of course, already seen their commercial analogues in ablative antifouling paints [58], as well as in diverse applications such as removable (“peel-off” or “tear-off”) layers on automotive or bicycle racing visors. Ablative coatings can be combined with other cleaning methods for very effective antifouling coatings, e.g., Tribou and Swain have reported the performance of static copper ablative coatings in combination with mechanical grooming as a means of maintaining the coatings in an operational condition for a period of six years [59]. Chemical modification of surfaces is another important strategy here. Other strategies such as nitric oxide production, use of peptoids that mimic protein-repellent peptides, zwitterionic functionalities found in membrane structures, and catechol functionalities used by mussels to immobilize poly(ethylene glycol) have recently been comprehensively reviewed [60].

### 2.2. Surface Texture Control and Biomimetics

Antifouling strategies, methods, or materials that are non-toxic, or at least have little widespread impact upon non-target organisms outside of the surface to be protected, are very attractive from an environmental protection perspective, and many non-toxic antifouling methods have been examined with this in mind [61]. Antifouling surfaces that rely on engineering a specific surface topography or texture into a material or coating have been widely examined for potential antifouling aspects, and these have recently been comprehensively reviewed by Carve et al. [62]. Multiple commercial applications have considered this approach, with some promising technologies in development or at commercial stage (see for example Finsulate: www.finsulate.com—accessed on 1 May 2020). Many of these have taken inspiration from nature and have incorporated aspects of sharkskin; crustaceans; or, more recently, mucus generation and hydrophilic hierarchical micro/nano-structures found on marine organisms [63]. Ren et al. have recently reported “mucus-like and hierarchical ciliary bionic” antifouling surfaces for marine antifouling applications [63]. Another group have used snail shells exhibiting oleophobic properties and a surface texture [64] to explore the feasibility of recreating similar structures on the inner surfaces of conventional biliary stents for antifouling purposes in medical fields [65]. Detailed electron microscopy observations of snail shells demonstrated surface features of around 200 nm in size. The authors hypothesized that when water enters the pores between these surface features, a film of water exhibiting super-nanohydrophilic structure forms on the shell, thus creating an oil-repellent surface with some antifouling effect. They were able to replicate analogous surface features on stents and conduct in vivo studies that demonstrated antifouling effects on the basis of a reduction in fouled surface area observed [65].

Erramilli and Genzer systematically reviewed the attributes of published surface topographies with antifouling or self-cleaning properties [66]. Decomposing the results of a range of published surface topographies on the basis of feature dimensions, geometry, and stiffness, allowed examination of the influence of different attributes of surface features on settlement or adhesion of both natural fouling organisms and synthetic particles. Many of the selected surface topographies were bioinspired or biomimetic, coming from sharkskin or from butterfly wing microstructure, for example. They noted general observations such as the influence of feature size on contact area between the particle and surface. They also noted that the stiffness plays an important role in governing the adhesive properties of the surface, concluding that individually or in conjunction, surface topography can act to affect the settlement of both natural organisms and synthetic particles on surfaces, but that further work was needed. Crucially, from a technology perspective, they highlighted that fabrication of functional topographies with high complexity in size and/or geometry of features is difficult (or perhaps prohibitively expensive) with the currently widely available tools, and that this hinders the creation (at least at scale) of complex hierarchical topographies—those most often observed in nature.

Carve et al., in their recent systematic review of the published effects of surface texture on marine biofouling, characterized key research methodologies [62]. Again, much of the published data concern surface textures that were inspired by or mimicked natural surfaces, even if the resulting surfaces were again often, by limitations enforced by fabrication technologies, quite removed from the original natural surfaces that served as inspiration. Carve et al. found that textures had no effect, or an inconclusive effect, on fouling in 46% of examined cases. It was notable that the ratios of feature height, width, or pitch to the body length of a settling organism were significant influences, and further research was recommended on hierarchical texture designs, as well as field studies in order to ground-truth laboratory results, some of which indeed has been attempted for more simplistic topographic designs [67]. Wen et al. recently reviewed biomimetic polymeric superhydrophobic surfaces and nanostructures from their fabrication to current and potential applications [68]. Some of the reviewed surfaces also have reported antifouling effects, although, as pointed out by the authors, “surfaces with poor physical and chemical properties are generally unable to withstand the severe conditions of the outside world; thus, it is necessary to optimize the performances of such materials to yield durable superhydrophobic surfaces” [68].

**Figure 4:**
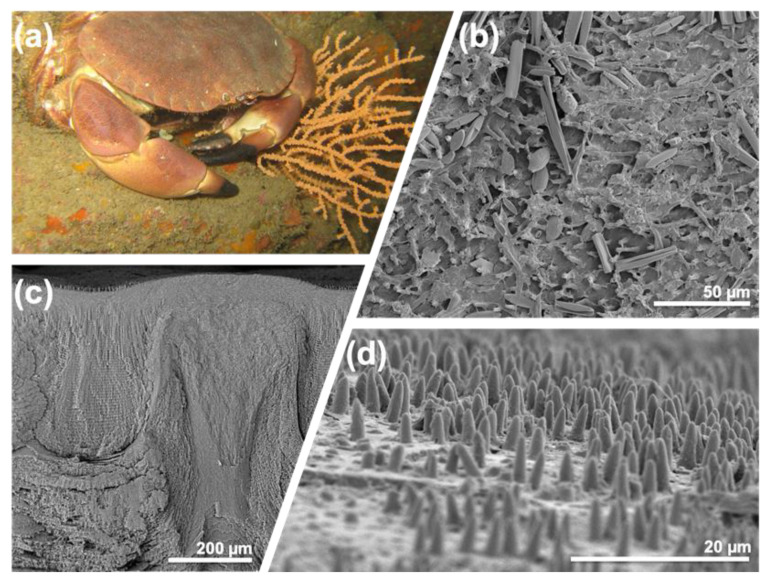
Crustaceans continue to be a source of inspiration in the search for new antifouling strategies and other technologies such as nanostructured composites. Here, electron microscopy images of the surface of the carapace of the marine decapod crustacean *Cancer pagurus* (**a**), also showing laminar structure in cross-section (**c**, scale bar = 200 µm) show the presence of micro-topographic features presented to any colonising organisms (epibionts). Many of the upper and under surfaces of *C. pagurus* are covered in these micro-scale spines (microtrichia), approximately some 5 to 20 microns in length (**d**, scale bar = 20 µm). These surfaces do sustain some colonisation (in this case, benthic diatoms species) (**b**, scale bar = 50 µm); however, the role and extent of any natural antifouling provided by these surface structures against larger epibionts (particularly other calcareous colonising species such as polychaetes) are not yet fully understood. Image (**a**) by Matthieu Sontag from Wikimedia Commons, licensed under CC BY-SA 4.0.

Brzozowksa et al., inspired by the marine decapod crustacean *Myomenippe hardwickii*, designed hierarchical surface microtopographies that replicated the critical features observed on the crustacean surface [69], while others [70] have examined some of the characteristics of the micro-structures from *Cancer pagurus* (Figure 4). The micropatterned surfaces of Brzozowska et al. were modified with zwitterionic polymer brushes or with polyelectrolyte multilayers (using layer-by-layer approaches) to enhance their antifouling and/or fouling-release potential. Zhao et al. recently reported another interesting approach in which the microstructure surface of *Laminaria japonica* was reproduced using a moulding process and was prepared by layer-by-layer assembly [71], while Fu et al. proposed a topography to combat biofouling by using a surface with microscopic ridge-like morphology inspired by the leaves of the mangrove tree, *Sonneratia apetala* [72]. Meanwhile, Rozenzweig et al. recently demonstrated biomimetic nano-pillars that inhibit eukaryotic filamentous fungal growth and possess fungicidal properties [73].

### 2.3. Progress in Biomimetic Sharkskin Surfaces

**Figure 5:**
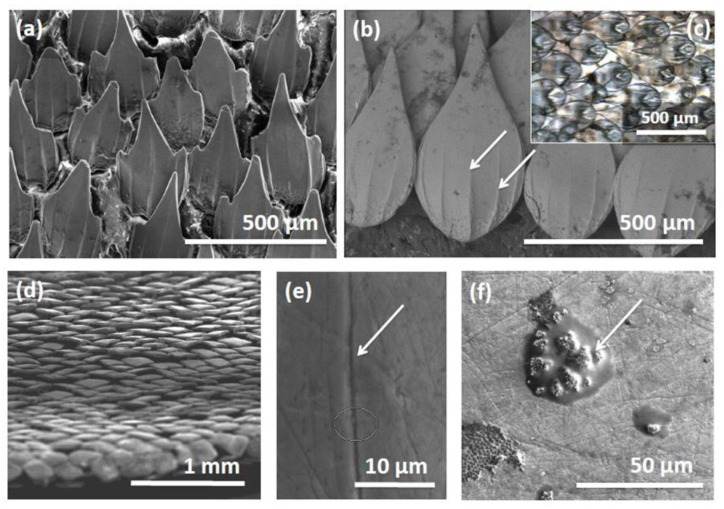
The shape and structure of dermal denticles from the small catshark, *Scyliorhinus canicula* (examined using electron microscopy). ((**a**,**b**) scale bar = 500 µm) Variations in the shape of dermal denticles observed from skin samples in different body locations in one individual specimen of this species. Similar structures to the microscopic ridges on the surface of individual denticles (as indicated by arrows in (**b**), scale bar = 500 µm) are thought to be responsible for drag reduction in sharks. It is interesting to observe the degree of overlap between denticles (inset (**c**), scale bar = 500 µm) and how the denticles extend and completely envelope the shark, including the trailing edges of fin surfaces (**d**), scale bar = 1 m)m. The role these structures play in both drag reduction and in antifouling is still of keen research interest. An interesting observation from close examination of denticle surfaces in *S. canicula* is that the upper surface of individual denticles are often scarred and grooved with microscopic ridges (**e**), scale bar = 10 µm) perhaps from contact with other sharks or other behaviours that remove attached organisms, although fouling may be observed in some regions (**f**) scale bar = 50 µm.

Sharkskin (Figure 5) is another natural surface that is now well known as providing inspiration in the search for a number of much-vaunted technologies, particularly those associated with the search for drag-reducing surfaces, and more recently, antifouling surface textures (perhaps in tandem with drag reduction) [74,75,76]. Perhaps the defining features of sharkskin are the dermal denticles, which protrude from the skin of sharks (Figure 5), features that make these surfaces so intriguing from a research perspective. A number of recent studies have demonstrated that the potential still exists for sharkskin to provide inspiration in designing new technologies; however, as recently pointed out by Domel et al., when providing new design guidelines for the production of low-drag coatings for aquatic and aerospace applications [77], shark denticles are complex in function and morphology and are still not entirely understood, despite extensive and intensive study. One interesting aspect of these denticles is their variation in size and shape between shark species (and within species, Figure 5); studies on denticle hydrodynamics have suggested that these structures reduce drag and increase both lift and thrust [77].

Chien et al., in a similar study to that by Sullivan et al. [78], recently investigated the microscale structure of denticles from skin samples at different body locations on a shark, analysing the roughness and wetting properties and evaluating the effect of the surface properties on bacterial attachment and biofilm formation [79]. The microscale structure was reported as not only affecting surface properties but also the biological attachment process, and the authors concluded that the microscale topography of sharkskin promoted bacterial attachment at an early stage but prevented bacteria from developing biofilms. Munther et al. generated placoid-scale patterns using micro-fabrication techniques and micro-moulding with an engineered height gradient to deter organism settlement [80]. Durability studies showed that the integrity of patterns was not easily compromised, and significant decreases in *Escherichia coli* settlement were observed when measuring the effectiveness of pristine patterns, although patterns were less effective when mechanical wear was apparent.

Arisoy et al. also used sharkskin as an inspiration to create bioinspired photocatalytic surfaces via nanoimprint lithography [75]. They reported that the textured surfaces created reduced the attachment of *Escherichia coli* by around 70% compared with smooth films with the same chemical composition. The authors also reported that patterned surfaces were fabricated using a solution-processable and roll-to-roll compatible technique, enabling the production of large-area, high-performance coatings at scale—a key point from a commercial perspective. Pu et al. created a biomimetic sharkskin using a polydimethylsiloxane-embedded elastomeric stamping method [81]. The antifouling properties of the biomimetic sharkskin surface with microstructures were reported as superior to a smooth surface using the same polymers as substrates. Moreover, the air layer fixed on the surface of the created material was determined to have a key role in anti-adhesion of potential fouling organisms. Other recent studies have examined various forms of biomimetic sharkskin to mitigate membrane biofouling for desalination applications [82], or have used functionalized polydimethylsiloxane (PDMS) membranes with sharkskin patterns for dressing applications (where the sharkskin pattern aids in forming a super-hydrophobic surface for inhibiting bacterial adhesion) [83].

### 2.4. Inspiration for Mechanical Grooming and Combined Antifouling Methods

Grooming and other mechanical means of cleaning can also be very important functions for organisms that live in environments in which surfaces can become easily contaminated, or aquatic environments where there is often a continuous supply of organisms looking to colonize available surfaces. These organisms (such as crustaceans) face the challenge that certain aspects of their external surfaces, for example their eyes and antennae, have to be kept clean in order to respond adequately to external signals. Many, therefore, have specialised mechanical cleaning mechanisms or specialised physical adaptions, which may be combined with other multi-functional strategies, such as micro- and nanostructured surfaces exhibiting anti-adhesive properties, in order to achieve this. This area seems ripe for biomimetic design, and yet does not feature prominently in the published literature concerning mechanical grooming or cleaning methods; further examination of the mechanisms of crustaceans and other organisms could enhance the design and efficiency of rotary cleaning brushes or wipers, for example. However, Liu et al. recently described a new biomimetic antifouling approach involving water jets that was inspired by marine kelp [84], which appeared to reduce settlement of benthic diatom species such as *Phaeodactylum tricornutum*. Improving the efficiency of both mechanical and water jet cleaning methods would appear to hold great promise in a combined antifouling strategy.

The use of atomic force microscopy to study the eye of crab *Carcinus maenasi* has also suggested potential antifouling properties of the microtopography of this surface [85], or it could perhaps be examined in terms of ease of cleaning rather than antifouling per se. In addition, most ocular systems seem well adapted to prevent bacterial infections, and although biofilm-associated infections of the human eye appear to have increased alongside the growth in use of ocular implants [86], the natural cleaning mechanisms of the eye would appear to be quite efficient. Improving the efficiency of both mechanical and water jet cleaning methods would appear to hold great promise in a combined antifouling strategy.

Perhaps there is an opportunity to combine such approaches with “smart coatings” that facilitate timely release for even more efficient cleaning [87]. For example, approaches such as on-demand liquid secretion [88] may prove useful in the release of natural products and could be combined with mechanical grooming methods for optimum surface cleaning. Other dynamic surfaces, such as the approach reported by Shivappoja et al. for actively and effectively detaching biofouling, would also appear to be interesting, and perhaps somewhat underexplored for antifouling applications [89].

A clear concept has emerged from the desire to move away from biocides towards more holistic approaches to antifouling—there is no “silver bullet” in many cases, and a multi-functional, multi-facetted approach can have many benefits; indeed, such a multi-functional approach is perhaps the “standard” approach in the natural world. Ralston and Swain have previously pointed out that improving the success of biomimetics for marine fouling control perhaps lies with identifying and reproducing natural strategies in a synergistic manner and in the context of the environment in which the organism lives rather taking any one factor (such as an aspect of surface topography) in isolation [90]. It is very clear that natural materials are rarely uniform and homogenous at the micro-scale; instead, the insect cuticle, sharkskin, or the epicuticular waxes of plants are often observed to have different material laminations or substructures for strength or multi-functionality [91], different roughness scales depending on location and function or other variations (see Figure 4 and Figure 5, above). This is contained in the “hierarchical structure of biological materials” [2] and contributes to the high degree of adaptability of natural systems. How to integrate these ideas with modern fabrication methods, coating development, and formulation remains a challenge if practical antifouling solutions are to be provided at scale.

## 3. Conclusions and Outlook

In conclusion, we offer the following observations and recommendations for future work in this area, on the basis of evident gaps in the current literature:

i. An environmentally conscious, biocide-free solution to biofouling is an attractive target, as it aligns with societal and political objectives. This is in contrast to the majority of commercially available solutions.
ii. Future research should take into account the plurality of natural processes achieving the same goal. For example, the “lotus effect” and “*Salvinia* effect” are two distinct hydrophobic strategies, with the latter better suited to submersible applications.
iii. Alongside antifouling effectiveness, other salient physicochemical properties of proposed biomimetic solutions should be ascertained, so as to better identify suitable applications. For instance, many proposed solutions lack the durability required for external applications.
iv. Topographical solutions are often beyond the economical limits of technology, despite many efficient structures existing in the natural world.
v. The successful scaling and integration of antifouling strategies into industry-standard processes is required to promote adoption of these solutions. However, this is, as of yet, an under-explored avenue.
vi. A multi-faceted approach perhaps holds the greatest promise of a widely applicable solution to biofouling.

This broad survey of recent literature, particularly that of the past decade, confirms again that biomimetic approaches to the development of antifouling technologies undoubtedly holds great promise, and research in this area is gathering pace, with many areas still to be explored. However, it would seem to be the case that commercial technologies resulting from these approaches often are yet to be realised from much of the reviewed research. Perhaps this is a result of a long development pipeline, or, perhaps more likely, there is still (as yet) an incomplete understanding of how natural surfaces self-clean or control fouling in many areas, and where the mechanisms are largely understood, there are often difficulties in faithfully replicating the complex natural structures or chemistries at sufficient scale for commercial application with the current technologies at hand.

The question remains as to what can be done to further advance or accelerate integration of biomimetic materials and bioinspired design into future viable and commercial antifouling technologies, or those other technological challenges mentioned at the beginning of this review. Perhaps it is again important to reiterate again that rather than merely “mimicking” existing natural solutions (by directly replicating and applying them in an analogous fashion to how they operate in the natural world), we should continue to strive to be truly “inspired” by natural materials and processes into new solutions for technological development.

## References

[1] Salta M., Wharton J.A., Stoodley P., Dennington S.P., Goodes L.R., Werwinski S., Mart U., Wood R.J.K., Stokes K.R. (2010). Designing biomimetic antifouling surfaces. Philos. Trans. R. Soc. A Math. Phys. Eng. Sci..

[2] Vincent J.F.V. (2009). Biomimetics—A review. Proc. Inst. Mech. Eng. Part H.

[3] Addadi L., Joester D., Nudelman F., Weiner S. (2006). Mollusk Shell Formation: A Source of New Concepts for Understanding Biomineralization Processes. Chem. Eur. J..

[4] Mora C., Tittensor D.P., Adl S., Simpson A.G.B., Worm B. (2011). How Many Species Are There on Earth and in the Ocean?. PLoS Biol..

[5] Gordon J.E. (1968). The New Science of Strong Materials: Or Why You Don’t Fall Through the Floor.

[6] Bhushan B. (2009). Biomimetics: Lessons from nature–an overview. Philos. Trans. R. Soc. A Math. Phys. Eng. Sci..

[7] Bar-Cohen Y. (2006). Biomimetics: Biologically Inspired Technologies.

[8] Flemming H.-C. (2020). Biofouling and me: My Stockholm syndrome with biofilms. Water Res..

[9] Bixler G.D., Bhushan B. (2012). Biofouling: Lessons from nature. Philos. Trans. R. Soc. A Math. Phys. Eng. Sci..

[10] Kim W., Tengra F.K., Young Z., Shong J., Marchand N., Chan H.K., Pangule R.C., Parra M., Dordick J.S., Plawsky J.L. (2013). Spaceflight Promotes Biofilm Formation by Pseudomonas aeruginosa. PLoS ONE.

[11] Sharahi J.Y., Azimi T., Shariati A., Safari H., Tehrani M.K., Hashemi A. (2019). Advanced strategies for combating bacterial biofilms. J. Cell. Physiol..

[12] Schultz M.P., Bendick J.A., Holm E.R., Hertel W.M. (2011). Economic impact of biofouling on a naval surface ship. Biofouling.

[13] Lacoursière-Roussel A., Bock D.G., Cristescu M.E., Guichard F., McKindsey C.W. (2016). Effect of shipping traffic on biofouling invasion success at population and community levels. Biol. Invasions.

[14] Soto-Giron M.J., Rodriguez-R L.M., Luo C., Elk M., Ryu H., Hoelle J., Santo Domingo J.W., Konstantinidis K.T. (2016). Biofilms on Hospital Shower Hoses: Characterization and Implications for Nosocomial Infections. Appl. Environ. Microbiol..

[15] Stewart P.S., William Costerton J. (2001). Antibiotic resistance of bacteria in biofilms. Lancet.

[16] Thomas K.V., Fileman T.W., Readman J.W., Waldock M.J. (2001). Antifouling Paint Booster Biocides in the UK Coastal Environment and Potential Risks of Biological Effects. Mar. Pollut. Bull..

[17] Thomas K.V., Brooks S. (2010). The environmental fate and effects of antifouling paint biocides. Biofouling.

[18] McNeil E.M. (2018). Antifouling: Regulation of biocides in the UK before and after Brexit. Mar. Policy.

[19] Nunes S.P. (2020). Can fouling in membranes be ever defeated?. Curr. Opin. Chem. Eng..

[20] Kyei S.K., Darko G., Akaranta O. (2020). Chemistry and application of emerging ecofriendly antifouling paints: A review. J. Coat. Technol. Res..

[21] De Nys R., Steinberg P.D., Willemsen P., Dworjanyn S.A., Gabelish C.L., King R.J. (1995). Broad spectrum effects of secondary metabolites from the red alga *delisea pulchra* in antifouling assays. Biofouling.

[22] Park J.S., Ryu E.-J., Li L., Choi B.-K., Kim B.M. (2017). New bicyclic brominated furanones as potent autoinducer-2 quorum-sensing inhibitors against bacterial biofilm formation. Eur. J. Med. Chem..

[23] Karnjana K., Soowannayan C., Wongprasert K. (2019). Ethanolic extract of red seaweed Gracilaria fisheri and furanone eradicate Vibrio harveyi and Vibrio parahaemolyticus biofilms and ameliorate the bacterial infection in shrimp. Fish Shellfish Immunol..

[24] Karnjana K., Nobsathian S., Soowannayan C., Zhao W., Tang Y.-J., Wongprasert K. (2020). Purification and Evaluation of N-benzyl Cinnamamide from Red Seaweed Gracilaria fisheri as an Inhibitor of Vibrio harveyi AI-2 Quorum Sensing. Mar. Drugs.

[25] Gao L., McCarthy T.J. (2006). The “Lotus Effect” Explained: Two Reasons Why Two Length Scales of Topography Are Important. Langmuir.

[26] Marmur A. (2004). The Lotus Effect: Superhydrophobicity and Metastability. Langmuir.

[27] Patankar N.A. (2004). Mimicking the Lotus Effect: Influence of Double Roughness Structures and Slender Pillars. Langmuir.

[28] Pereira J. (2017). Lotus. Effect Washing Machine. U.S. Patent.

[29] Zouaghi S., Bellayer S., Thomy V., Dargent T., Coffinier Y., Andre C., Delaplace G., Jimenez M. (2019). Biomimetic surface modifications of stainless steel targeting dairy fouling mitigation and bacterial adhesion. Food Bioprod. Process..

[30] Barthlott W., Schimmel T., Wiersch S., Koch K., Brede M., Barczewski M., Walheim S., Weis A., Kaltenmaier A., Leder A. (2010). The Salvinia Paradox: Superhydrophobic Surfaces with Hydrophilic Pins for Air Retention Under Water. Adv. Mater..

[31] Barthlott W., Mail M., Bhushan B., Koch K. (2017). Plant Surfaces: Structures and Functions for Biomimetic Innovations. Nano-Micro Lett..

[32] Babu D.J., Mail M., Barthlott W., Schneider J.J. (2017). Superhydrophobic Vertically Aligned Carbon Nanotubes for Biomimetic Air Retention under Water (*Salvinia* Effect). Adv. Mater. Interfaces.

[33] Zhou K., Li D., Xue P., Wang P., Zhao Y., Jin M. (2020). One-step fabrication of Salvinia-inspired superhydrophobic surfaces with High adhesion. Colloids Surf. A Physicochem. Eng. Asp..

[34] Busch J., Barthlott W., Brede M., Terlau W., Mail M. (2019). Bionics and green technology in maritime shipping: An assessment of the effect of Salvinia air-layer hull coatings for drag and fuel reduction. Philos. Trans. R. Soc. A: Math. Phys. Eng. Sci..

[35] Liang C., Ye Z., Xue B., Zeng L., Wu W., Zhong C., Cao Y., Hu B., Messersmith P.B. (2018). Self-Assembled Nanofibers for Strong Underwater Adhesion: The Trick of Barnacles. ACS Appl. Mater. Interfaces.

[36] Liang C., Strickland J., Ye Z., Wu W., Hu B., Rittschof D. (2019). Biochemistry of Barnacle Adhesion: An Updated Review. Front. Mar. Sci..

[37] Almeida M., Reis R.L., Silva T.H. (2020). Marine invertebrates are a source of bioadhesives with biomimetic interest. Mater. Sci. Eng. C.

[38] Basu S., Hanh B.M., Isaiah Chua J.Q., Daniel D., Ismail M.H., Marchioro M., Amini S., Rice S.A., Miserez A. (2020). Green biolubricant infused slippery surfaces to combat marine biofouling. J. Colloid Interface Sci..

[39] Zhang X., Liu H., Yue L., Bai Y., He J. (2020). Mussel-mimetic polymer underwater adhesives with l-Dopa functionality: Influencing adhesion properties and simplified operation procedures. J. Mater. Sci..

[40] Meng F., Liu Q., Wang X., Tan D., Xue L., Barnes W.J.P. (2019). Tree frog adhesion biomimetics: Opportunities for the development of new, smart adhesives that adhere under wet conditions. Philos. Trans. R. Soc. A Math. Phys. Eng. Sci..

[41] Nir S., Reches M. (2016). Bio-inspired antifouling approaches: The quest towards non-toxic and non-biocidal materials. Curr. Opin. Biotechnol..

[42] Bus T., Dale M.L., Reynolds K.J., Bastiaansen C.W.M. (2020). Thermoplastic, rubber-like marine antifouling coatings with micro-structures via mechanical embossing. Biofouling.

[43] Kommeren S., Guerin A.J., Dale M.L., Ferguson J., Lyall G., Reynolds K.J., Clare A.S., Bastiaansen C.W.M., Sullivan T. (2019). Sullivan Antifouling and Fouling-Release Performance of Photo-Embossed Fluorogel Elastomers. J. Mar. Sci. Eng..

[44] Scardino A.J., Hudleston D., Peng Z., Paul N.A., de Nys R. (2009). Biomimetic characterisation of key surface parameters for the development of fouling resistant materials. Biofouling.

[45] Halvey A.K., Macdonald B., Dhyani A., Tuteja A. (2019). Design of surfaces for controlling hard and soft fouling. Philos. Trans. R. Soc. A: Math. Phys. Eng. Sci..

[46] Selim M.S., Shenashen M.A., Elmarakbi A., Fatthallah N.A., Hasegawa S., El-Safty S.A. (2017). Synthesis of ultrahydrophobic and thermally stable inorganic–organic nanocomposites for self-cleaning foul release coatings. Chem. Eng. J..

[47] Xu Q., Zhang W., Dong C., Sreeprasad T.S., Xia Z. (2016). Biomimetic self-cleaning surfaces: Synthesis, mechanism and applications. J. R. Soc. Interface.

[48] Gorb E.V., Gorb S.N. (2017). Anti-adhesive effects of plant wax coverage on insect attachment. J. Exp. Bot..

[49] Hellio C., Thomas-Guyon H., Culioli G., Piovettt L., Bourgougnon N., Le Gal Y. (2001). Marine antifoulants from *bifurcaria bifurcata* (phaeophyceae, cystoseiraceae) and other brown macroalgae. Biofouling.

[50] Sánchez-Lozano I., Hernández-Guerrero C.J., Muñoz-Ochoa M., Hellio C. (2019). Biomimetic Approaches for the Development of New Antifouling Solutions: Study of Incorporation of Macroalgae and Sponge Extracts for the Development of New Environmentally-Friendly Coatings. Int. J. Mol. Sci..

[51] Pan J., Xie Q., Chiang H., Peng Q., Qian P.-Y., Ma C., Zhang G. (2020). “From the Nature for the Nature”: An Eco-Friendly Antifouling Coating Consisting of Poly(lactic acid)-Based Polyurethane and Natural Antifoulant. ACS Sustain. Chem. Eng..

[52] Myles A., Haberlin D., Esteban-Tejeda L., Angione M.D., Browne M.P., Hoque M.K., Doyle T.K., Scanlan E.M., Colavita P.E. (2018). Bioinspired Aryldiazonium Carbohydrate Coatings: Reduced Adhesion of Foulants at Polymer and Stainless Steel Surfaces in a Marine Environment. ACS Sustain. Chem. Eng..

[53] Mayombo N., Majewska R., Smit A. (2019). Diatoms associated with two South African kelp species: *Ecklonia maxima* and *Laminaria pallida*. Afr. J. Mar. Sci..

[54] Sullivan T. (2019). Cell Shape and Surface Colonisation in the Diatom Genus Cocconeis—An Opportunity to Explore Bio-Inspired Shape Packing?. Biomimetics.

[55] Wanka R., Finlay J.A., Nolte K.A., Koc J., Jakobi V., Anderson C., Clare A.S., Gardner H., Hunsucker K.Z., Swain G.W. (2018). Fouling-Release Properties of Dendritic Polyglycerols against Marine Diatoms. ACS Appl. Mater. Interfaces.

[56] Lachnit M., Buhmann M.T., Klemm J., Kröger N., Poulsen N. (2019). Identification of proteins in the adhesive trails of the diatom *Amphora coffeaeformis*. Philos. Trans. R. Soc. B Biol. Sci..

[57] Barthel D., Wolfrath B. (1989). Tissue sloughing in the sponge Halichondria panicea: A fouling organism prevents being fouled. Oecologia.

[58] Almeida E., Diamantino T.C., de Sousa O. (2007). Marine paints: The particular case of antifouling paints. Prog. Org. Coat..

[59] Tribou M., Swain G. (2017). The effects of grooming on a copper ablative coating: A six year study. Biofouling.

[60] Damodaran V.B., Murthy N.S. (2016). Bio-inspired strategies for designing antifouling biomaterials. Biomater. Res..

[61] Nurioglu A.G., Esteves A.C.C., de With G. (2015). Non-toxic, non-biocide-release antifouling coatings based on molecular structure design for marine applications. J. Mater. Chem. B.

[62] Carve M., Scardino A., Shimeta J. (2019). Effects of surface texture and interrelated properties on marine biofouling: A systematic review. Biofouling.

[63] Ren X., Guo M., Xue L., Zeng Q., Gao X., Xin Y., Xu L., Li L. (2020). A Self-Cleaning Mucus-like and Hierarchical Ciliary Bionic Surface for Marine Antifouling. Adv. Eng. Mater..

[64] Nishino T., Tanigawa H., Sekiguchi A. (2018). Antifouling Effect on Biomimetic Metamaterial Surfaces. J. Photopol. Sci. Technol..

[65] Sekiguchi A., Nishino T., Aikawa M., Matsumoto Y., Minami H., Tokumaru K., Tsumori F., Tanigawa H. (2019). The Study of Bile Duct Stent Having Antifouling Properties Using Biomimetics Technique. J. Photopol. Sci. Technol..

[66] Erramilli S., Genzer J. (2019). Influence of surface topography attributes on settlement and adhesion of natural and synthetic species. Soft Matter.

[67] Sullivan T., Regan F. (2017). Marine diatom settlement on microtextured materials in static field trials. J. Mater. Sci..

[68] Wen G., Guo Z., Liu W. (2017). Biomimetic polymeric superhydrophobic surfaces and nanostructures: From fabrication to applications. Nanoscale.

[69] Brzozowska A.M., Parra-Velandia F.J., Quintana R., Xiaoying Z., Lee S.S., Chin-Sing L., Jańczewski D., Teo S.L.-M., Vancso J.G. (2014). Biomimicking micropatterned surfaces and their effect on marine biofouling. Langmuir.

[70] Sullivan T., McGuinness K., Connor N.E.O., Regan F. (2014). Characterization and anti-settlement aspects of surface micro-structures from *Cancer pagurus*. Bioinspir. Biomim..

[71] Zhao L., Chen R., Lou L., Jing X., Liu Q., Liu J., Yu J., Liu P., Wang J. (2020). Layer-by-Layer-Assembled antifouling films with surface microtopography inspired by Laminaria japonica. Appl. Surf. Sci..

[72] Fu J., Zhang H., Guo Z., Feng D., Thiyagarajan V., Yao H. (2018). Combat biofouling with microscopic ridge-like surface morphology: A bioinspired study. J. R. Soc. Interface.

[73] Rosenzweig R., Marshall M., Parivar A., Ly V.K., Pearlman E., Yee A.F. (2019). Biomimetic Nanopillared Surfaces Inhibit Drug Resistant Filamentous Fungal Growth. ACS Appl. Bio Mater..

[74] Dean B., Bhushan B. (2010). Shark-skin surfaces for fluid-drag reduction in turbulent flow: A review. Philos. Trans. R. Soc. A: Math. Phys. Eng. Sci..

[75] Dundar Arisoy F., Kolewe K.W., Homyak B., Kurtz I.S., Schiffman J.D., Watkins J.J. (2018). Bioinspired Photocatalytic Shark-Skin Surfaces with Antibacterial and Antifouling Activity via Nanoimprint Lithography. ACS Appl. Mater. Interfaces.

[76] Fu Y.F., Yuan C.Q., Bai X.Q. (2017). Marine drag reduction of shark skin inspired riblet surfaces. Biosurface Biotribology.

[77] Domel A.G., Domel G., Weaver J.C., Saadat M., Bertoldi K., Lauder G.V. (2018). Hydrodynamic properties of biomimetic shark skin: Effect of denticle size and swimming speed. Bioinspir. Biomim..

[78] Sullivan T., Regan F. (2011). The characterization, replication and testing of dermal denticles of *Scyliorhinus canicula* for physical mechanisms of biofouling prevention. Bioinspir. Biomim..

[79] Chien H.-W., Chen X.-Y., Tsai W.-P., Lee M. (2020). Inhibition of biofilm formation by rough shark skin-patterned surfaces. Colloids Surf. B Biointerfaces.

[80] Munther M., Palma T., Angeron I.A., Salari S., Ghassemi H., Vasefi M., Beheshti A., Davami K. (2018). Microfabricated Biomimetic placoid Scale-Inspired surfaces for antifouling applications. Appl. Surf. Sci..

[81] Pu X., Li G., Huang H. (2016). Preparation, anti-biofouling and drag-reduction properties of a biomimetic shark skin surface. Biol. Open.

[82] Choi W., Lee C., Lee D., Won Y.J., Lee G.W., Shin M.G., Chun B., Kim T.-S., Park H.-D., Jung H.W. (2018). Sharkskin-mimetic desalination membranes with ultralow biofouling. J. Mater. Chem. A.

[83] Lin Y.-T., Ting Y.-S., Chen B.-Y., Cheng Y.-W., Liu T.-Y. (2020). Bionic shark skin replica and zwitterionic polymer brushes functionalized PDMS membrane for anti-fouling and wound dressing applications. Surf. Coat. Technol..

[84] Liu G., Yuan Z., Incecik A., Leng D., Wang S., Li Z. (2020). A new biomimetic antifouling method based on water jet for marine structures. Proc. Inst. Mech. Eng. Part M J. Eng. Marit. Environ..

[85] Greco G., Lanero T.S., Torrassa S., Young R., Vassalli M., Cavaliere A., Rolandi R., Pelucchi E., Faimali M., Davenport J. (2013). Microtopography of the eye surface of the crab *Carcinus maenas*: An atomic force microscope study suggesting a possible antifouling potential. J. R. Soc. Interface.

[86] Bispo P., Haas W., Gilmore M. (2015). Biofilms in Infections of the Eye. Pathogens.

[87] O’Neill P., Barrett A., Sullivan T., Regan F., Brabazon D. (2016). Rapid Prototyped Biomimetic Antifouling Surfaces for Marine Applications. Mater. Today Proc..

[88] Gelebart A.H., Liu D., Mulder D.J., Leunissen K.H.J., van Gerven J., Schenning A.P.H.J., Broer D.J. (2018). Photoresponsive Sponge-Like Coating for On-Demand Liquid Release. Adv. Funct. Mater..

[89] Shivapooja P., Wang Q., Orihuela B., Rittschof D., López G.P., Zhao X. (2013). Bioinspired Surfaces with Dynamic Topography for Active Control of Biofouling. Adv. Mater..

[90] Ralston E., Swain G. (2009). Bioinspiration—The solution for biofouling control?. Bioinspir. Biomim..

[91] Han Z., Mu Z., Yin W., Li W., Niu S., Zhang J., Ren L. (2016). Biomimetic multifunctional surfaces inspired from animals. Adv. Colloid Interface Sci..

